# Inputs of Total and Labile Dissolved Metals from Six Facilities Continuously Discharging Treated Wastewaters to the Marine Environment of Gran Canaria Island (Canary Islands, Spain)

**DOI:** 10.3390/ijerph182111582

**Published:** 2021-11-04

**Authors:** Marta Rodrigo Sanz, Vanessa Millán Gabet, Jean-Louis Gonzalez

**Affiliations:** 1 Water Department, Instituto Tecnológico de Canarias (ITC), Pozo Izquierdo, s/n, 35019 Santa Lucía, Spain; 2 Unit of Biogeochemistry and Ecotoxicology, Institut Français de Recherche pour l’Exploitation de la Mer (IFREMER), 83507 La Seyne-sur-Mer, France; Jean.Louis.Gonzalez@ifremer.fr

**Keywords:** wastewater, monitoring, heavy metals, DGT, discharges, water quality

## Abstract

The presence of ten metals (Cd, Ni, Pb, Cr, Cu, Zn, Al, Fe, Mn, and Co) was investigated in the final discharge of six facilities, including four wastewater treatment plants, which were continuously discharging treated wastewater to the coastal environment in Gran Canaria Island. A four-day sampling campaign was carried out at each facility in July 2020, in which both the spot samplings technique and the diffusive gradient in thin-film technique (DGT) were carried out to measure total dissolved metals and the in situ labile metal fraction, respectively. After the necessary sample preparation steps, measurements were carried out by ICP-MS for both samplings. Raw data referred to the spot total dissolved and DGT-labile metal concentrations were reported. In general, the average metal concentrations were dispersed in a broad range. As expected, the highest metal contents were found in those facilities with larger industrial contributions. The values of annual average environmental quality standards (AA-EQS) were used to assess the total dissolved metal concentrations for every metal in every final discharge. In only one of the studied facilities, some metals (Ni and Zn) exceeded these EQS within the receiving waterbody, highlighting the need for more efficient treatment targeted towards a specific discharging-water quality. In addition, the total dissolved and labile metal daily fluxes of discharge were calculated to estimate the contribution of every effluent to the receiving water bodies.

## 1. Introduction

A significant proportion of metals enter marine water bodies via surface runoff and municipal or industrial wastewater discharges, which are important secondary sources of these substances. Indeed, wastewater treatment plants (WWTP), due to their high continuous flow rates, discharge important amounts of trace metals and other contaminants into the marine environment, which cannot be fully retained in these facilities [1,2,3]. Heavy metals are among the priority pollutants (in particular, Cd, Ni, Pb, and Hg) of major concern due to their toxic effect and long-term accumulation in sediments and aquatic organisms [4,5]. Furthermore, other types of facilities also continuously discharge treated wastewaters with trace metals to the sea, such as thermal power plants, marine aquaculture, and desalination plants, etc. [6].

Coastal seawater is one of the most important water resources in the Canary Islands. Its quality not only impacts tourism, which is the main economic activity, but also the seawater intake of desalination plants that provide water for human consumption to most of the island’s inhabitants, particularly in the case of Gran Canaria. Thus, the deterioration of seawater quality is crucial and critical.

The EU (European Union) Policy on Water Protection is based on a combined approach in which the emission limit value (ELV) and the water quality objectives (WQO) are mutually reinforcing. The ELV approach focuses on the maximum allowable quantities of pollutants discharged from a particular source into the aquatic environment.

Three regions in Spain have developed regional regulatory limits on the metals’ levels in the discharges to the sea: Cantabria, País Vasco, and Andalucía. They have set different limits for 15 metals and metalloids to prevent environmental risks from both industrial wastewater and sewage, including total mercury with an ELV ranging from 2.4 to 100 µg L^−1^, total arsenic [500–1200 µg·L^−1^], total cadmium [14–400 µg·L^−1^], total chromium [360–3000 µg·L^−1^], hexavalent chromium [36–500 µg·L^−1^], total nickel [720–5000 µg·L^−1^], total lead [200–500 µg·L^−1^], total aluminum [3000–10,000 µg·L^−1^], total copper [500–3000 µg·L^−1^], total zinc [1800–10,000 µg·L^−1^], total tin [500–20,000 µg·L^−1^], total manganese [2000–10,000 µg·L^−1^], iron [2000–3600 µg·L^−1^], total selenium [50–200 µg·L^−1^], and total titanium [1000–5000 µg·L^−1^].

However, the regional regulatory limits on the metals’ levels in the discharges to the sea are not currently defined, even though the competent Canarian regulatory body is working on them. Consequently, the requirements for the discharge of metals (and other substances) discharged from any facility that discharges treated wastewater to the sea must be strictly controlled. These requirements must comply with the coastal WQO, particularly with the Environmental Quality Standards (EQS) for metals, to effectively protect water bodies and ensure their “good chemical status”. The chemical status assessment is used alongside the ecological status assessment to determine the overall quality of a water body [7]. In the EU countries, the Water Framework Directive (WFD) implementation results in a challenge due to the removal of metals in wastewater discharges [8]. In its latest upgrade (EC 2013, Annex I) [9], four metals (Cd, Hg, Ni, and Pb) were classified as priority substances. After being transposed to the Spanish regulation (Real Decreto 817/2015) [10], Cu, Cr VI, and Zn were also included as preferable substances in surface waters, as well as the metalloids As and Sb.

Metals can occur in the WWTPs as different physico-chemical “species”. Metals may be found in different forms, including solutions, colloids or suspensions, made up by both simple ions (free ions) or particles and complex organic or inorganic compounds [11]. During wastewater treatments, the speciation for a given metal may be modified due to degradation of organic ligands, biomass uptake, and changes in pH [2]. Although mechanisms of heavy metal removal during primary settling are not fully understood [3], it has been assumed that most of the metals are significantly removed from the final effluents in conventional activated sludge WWTPs. This removal results mainly from the metals partitioning to the solid phase of the treatment systems [12]. The removal rate in the different treatment plants may be affected by many factors, including the type of metal, concentration in the influent, interactions with microbes in the sewage treatment system, and the treatment processes [2,12]. Metal removal in WWTPs often requires additional tertiary treatment, such as chemical precipitation, oxidation, or coagulation techniques [3,13].

Reported data indicate that dissolved metals in WWTPs are less efficiently removed, becoming the main fraction in treated effluents and causing an enrichment on the receiving water bodies [12,14,15]. The removal of the labile and dissolved fractions of some metals is highly variable and is not always efficient. In fact, the labile metal concentrations (when measurable) have been reported to remain nearly unchanged [16,17,18] during treatment. Thus, the assessment of this variability involves many operationally inherent factors such as recirculation, the use of chemicals or reagents during flocculation, and other tertiary wastewater treatments.

DGT devices have been reported to provide accurate time-weighted average concentrations of dissolved labile metals in wastewaters [12,16,17,19,20,21]. Thus, such devices are appropriate tools to assess in situ metal pollution in wastewater, minimizing the analytical chemistry challenge related to the temporal variability and chemical complexity of this matrix. In addition, during in situ DGT deployment, dissolved labile metals are pre-concentrated, so lower concentrations of this fraction can be measured. The benefit of using diffusive gradient in thin-films (DGT) technique for monitoring purposes [22] is that they measure labile species of trace metals. DGT-labile metal concentrations may represent the bioavailable metal fraction more accurately than filterable metal or total metal [23,24]. Therefore, it has been suggested that DGT-labile metal concentrations might represent the “potentially bioavailable” metal fraction to the biota [25,26].

Although the use of DGTs, as described above, can be advantageous, the assessment of metals by the direct deployment of DGTs in the wastewater discharge pipeline has not been reported in many studies in the literature [17,20]. On the contrary, some studies deployed the DGTs within wastewater samples at the laboratory, under stirring and temperature-and-time-controlled conditions [12,16,19,21].

In this study, we aimed to measure the dissolved and labile concentrations of ten metals in the final effluents of six facilities (including four WWTP with different wastewater intakes) that continuously discharged into the coastal receiving water bodies of the Island of Gran Canaria. Based on these results, the total dissolved metal concentrations for every metal and effluent would be assessed considering the values of annual average environmental quality standards (AA-EQS). In addition, the total dissolved and labile metal daily fluxes discharges would be calculated to estimate the contribution of each effluent to the receiving water bodies.

## 2. Materials and Methods

### 2.1. Sampling Facilities

**Figure 1:**
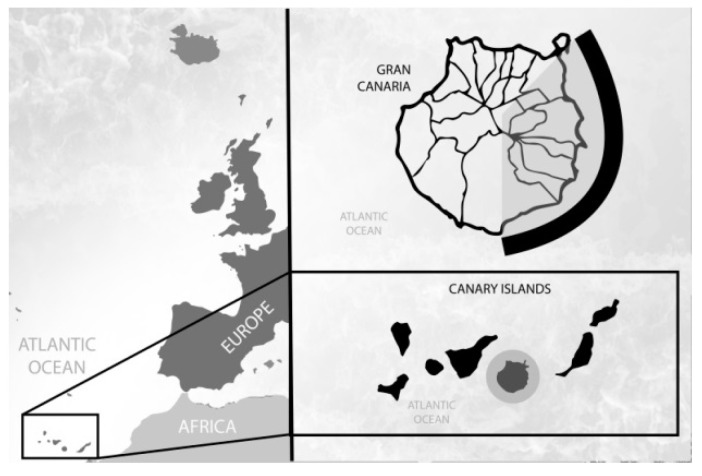
Location of the area where the studied facilities are situated.

Gran Canaria is in the middle of the Canary Islands archipelago in the Atlantic Ocean, southwest of Spain and northwest of Africa. All the facilities under study are located on the east coast of the island, where most of the population and economic activity are concentrated (Figure 1).

Six final discharges from different facilities were studied at the same time in July 2020. Several sources of wastewater and types of installations that continuously discharge into the sea were considered. They were chosen to ensure the representativeness of the existing WWTPs in the islands based on their capacity, their treatment technologies, and the diversity of the collected influents. Those finally selected were:

- TP-1: WWTP, which mainly collects household wastewater from the city of Las Palmas de Gran Canaria. This plant was considered as a reference for a typical large Canarian WWTP with domestic inputs.
- TP-2 and TP-3: medium size WWTP, which collects mixed household and industrial sewage inlets.
- TP-4: WWTP, which collects only industrial wastewater from two industrial areas of the island.
- TP-5: a coastal thermal power plant, which produces one of the largest discharges of cooling wastewater in the Canary Islands.
- TP-6: an indoor seawater aquaculture (fish) farm.

The main characteristics of these plants are summarised in Table 1.

The effluents (outlets) were discharged through underwater outfalls into the marine receiving water.

### 2.2. Sample Collection

All the material used in the field and in the laboratory was cleaned and then soaked in a 10% HNO_3_ (69%, ultrapure grade) acid bath overnight. Once rinsed thoroughly with ultrapure water (type I or better: ≥18 MΩ·cm resistivity), they were stored and sealed in clean plastic bags until being used [27].

- DGT-Deployment

We measured labile metals concentrations by using the DGT technique. All the used DGT devices were purchased from the same supplier (DGT^®^ Research Ltd., Lancaster, UK) and the same production batch.

At each facility, LSNM-NP for metals (cationic) DGT devices (0.8 mm agarose-diffusive layer, polyethersulphone 0.45 µm pore size filter membrane, and Chelex-100 binding-gel layer) were in situ deployed during 4 completed days (4 d × 24 h). DGT devices were deployed in triplicate, freely suspended at the head-chamber of each underwater outfall, at about 30 cm below the surface (Supplementary Information Figure S1).

To prevent damage from side impacts in the header chamber, we joined the DGT devices to a plastic holder unit, protected with a nylon net. These DGT systems were assembled under a laminar flow hood just before every experiment.

After 4 complete days of exposure, the DGT devices were retrieved and sent to IFREMER laboratory, where the opening, Chelex-100 recovery, acid elution, and analysis were performed. Laboratory and field DGT blanks were used for controlling the potential contamination of the DGT samplers during their transport, handling for deployment, and processing.

- Spot-sampling

While the DGTs were deployed, at the same sampling point, we collected spot water samples with handheld samplers every 2 days: at DGT deployment, day 0; during DGT deployment, day 2; and at DGT retrieval, day 4.

Dissolved Organic Carbon (DOC), Suspended Particulate Matter (SPM), Turbidity (Turb), and the dissolved metal concentrations on the spot samples were determined at the laboratory.

- In situ parameters measurements

Simultaneously with the spot sampling, a calibrated YSI Pro DSS multiparameter probe was used for the effluent in situ readings of temperature (T), pH, electrical conductivity (EC, 25 °C), and dissolved oxygen (DO).

### 2.3. Sample Analyses

- Trace elements in DGTs by ICP-MS

Trace metals in DGTs were measured at the Unit of Biogeochemistry and Ecotoxicology laboratory of IFREMER, where DGT devices were dismantled, and the Chelex-100 resins were eluted in 1.25 mL of 1 M HNO_3_ acid solution (ultrapure grade nitric acid 65%, Merck Millipore, Germany, + ultrapure water) for at least 24 h at room temperature. The concentration of trace elements on the resulting acid extracts after 5-times dilution with ultrapure water (18.2 MΩ·cm) was determined by ICP-MS (Thermo iCAP Q, KED (He) mode). All reagents, standards, samples, and blanks were prepared using supra pure acids (HCl and HNO_3_) and previously cleaned LDPE (low-density polyethylene) or Teflon flasks.

- Trace elements in spot water samples by ICP-MS

The total dissolved concentrations (<0.45 µm) of trace metals in spot water samples were measured by ICP-MS by the Clinical and Analytical Toxicology Service (SERTOX) of the University of Las Palmas de Gran Canaria (ULPGC). Filtered water samples were analyzed using an Agilent 7900 ICP-MS (Agilent Technologies, Tokyo, Japan). The ICP-MS was equipped with standard nickel cones, MicroMist glass concentric nebulizer, and an Ultra High Matrix Introduction (UHMI) system.

Water samples were prepared for analysis based on [28] as follows: 130 μL of 0.45 µm filtered water, 1120 μL of nitric acid solution (2% nitric acid in ultrapure water), and 50 μL of internal standards (ISTD) until a final volume of 1.3 mL. ISTD solution was composed of scandium, germanium, rhodium, and iridium at a stock concentration of 5 mg·mL^−1^ each. Pure standards of elements in acid solution (5% HNO_3_, 100 mg·L^−1^) were purchased from CPA Chem (Stara Zagora, Bulgaria). A ten-point standard curve (0.005–20 µg·L^−1^) was prepared to contain all the elements included in the study.

### 2.4. Data Processing

- Treatment of DGT-labile-fraction metal concentration data

The in-situ DGT-labile metal concentration was calculated in two steps, as follows [29]:

- Calculation of the mass of metal (M), in g units, accumulated in the resin-gel layer, according to Equation (1):
  M = C_e_ ∗ (V_HNO3_ + V_gel_)/f_e_ (1)
  where:
  - C_e_ is the concentration of metals, in g·L^−1^ units, in the 1 M HNO_3_ elution solution
  - V_HNO3_ is the volume of HNO_3_ added to the resin gel (1.25 mL in this study)
  - V_gel_ is the volume of the resin gel (typically 0.15 mL)
  - f_e_ is the elution factor for each metal (typically 0.8)
- Calculation of the concentration of metal in water, in g·L^−1^ units, measured by the DGT device (C_DGT_), according to Equation (2):
  C_DGT_ = (M ∗ Δg)/(D ∗ t ∗ A) (2)
  where:
  - Δg is the thickness, in cm units, of the diffusive gel (approx. 0.08 cm) plus the thickness of the filter membrane (0.014 cm)
  - D is the diffusion coefficient of metal in the gel, available at [30]:
  - t is deployment time (in s units)
  - A is the exposure area (3.14 cm^2^)

For DGTs, the quantification limits for labile metals in the eluate solutions were 0.1 µg·L^−1^ for all the determined metals. When the concentrations were below this quantification limit, half of the quantification limit values were used for the calculations.

Exposed DGT-labile fraction metal concentrations were calculated as the average (mean value) of the concentrations measured in the three replicates. At the same time, the coefficients of variation were also calculated (in %). Values with coefficients of variation greater than 25% were used to identify and reject the outliers in the mean calculation.

- Treatment of Spot-sampling dissolved metal concentration data

The spot-sampling dissolved metal concentrations were calculated as the average (mean ± standard deviation (SD) values) of the concentrations measured in the 3 discrete samples collected on days 0, 2, and 4 of the DGT deployment. These were the quantification limits used (all in µg·L^−1^): 1.618·10^−4^ for Cd; 0.033 for Ni; 0.018 for Pb; 0.012 for Cr; 0.030 for Cu; 1.604 for Zn; 0.574 for Al; 0.415 for Fe; 0.001 for Mn, and 0.002 for Co. When the concentrations were below the respective quantification limit, half of the quantification limit values were used for the calculations.

Student’s *t*-test was used for establishing significant differences among independent results when necessary. Differences were statistically tested at the α = 0.05 significance level, corresponding to a confidence level of 95%. This analysis was carried out by the open-source Jamovi software (www.jamovi.org, accessed on 21 June 2021, version 1.6.9 (Sydney, Australia), and was specifically applied to:

- Verify that the mean concentrations of each metal in the exposed DGTs at each sampling site were higher than those in the DGTs blanks at the laboratory.
- Verify that, in the TP-6 results, the labile-fraction metal concentration (based on DGTs results, in triplicate) was higher than the total dissolved metal concentration (based on spot sampling results, in triplicate).

## 3. Results and Discussion

### 3.1. DGT Blanks

To monitor atmospheric contamination during assembling, transport, and deployment, or retrieval of the DGT devices, the metal content in DGT field blanks was also quantified. They all contained negligible concentrations of the determined metals. Furthermore, results in the DGT laboratory blanks were also significantly lower than all the 4-day exposed DGTs at each facility, except for Cu in TP-4 (treating industrial effluent). At this facility, the labile concentration of Cu determined in the discharged effluent was below the quantification limit.

As the DGT blank values (laboratory and field) were very low compared to those of the exposed DGTs, they were not subtracted when calculating the DGT-labile concentrations.

Supplementary Information S2 (Tables S1.1–S1.9) presents the statistical results.

### 3.2. Concentrations of Total Dissolved and Dissolved Labile Metals

Table 2 shows the concentration of total dissolved (in µg·L^−1^ units) and dissolved labile metals (in µg·L^−1^ units) measured in the final effluent from the 6 facilities. Note that the SD of the labile fraction has not been included in the table because the coefficients of variation were less than 25% in all cases. In order to approach the speciation of each metal, the percentage of the labile fraction is also shown.

#### 3.2.1. Total Dissolved Metals

We measured the total dissolved concentrations the 10 analysed metals in every sampled effluent, and the mean values of each facility fell among these ranges (all in μg·L^−1^ units): Cd [0.002–0.065], Ni [0.111–18.044], Pb [0.009–0.580], Cr [0.172–3.546], Cu [0.015–1.774], Zn [5.160–114.816], Al [0.786–697.056], Fe [0.299–9855.652], Mn [1.211–643.383], and Co [0.007–6.659].

As shown in Table 2, the mean concentration of every metal reached the highest values at the TP-4 facility, which exclusively treats industrial wastewater, except for Cu.

Currently, the TP-4 facility is treating 720 m^3^/day of wastewater from two major industrial areas on the island of Gran Canaria. The sources of this wastewater are small-to-medium industries, including, among others: a glass facility, paint industries, industrial laundries, automotive workshops, food industries, and a plastic processing factory. At this facility, dissolved mean concentrations of Al (697.056 ± 501.489 μg·L^−1^), Fe (9855.65 ± 4358.438 μg·L^−1^), and Mn (643.383 ± 233.195 μg·L^−1^) were high compared to the reported values for entirely or mixed industrial wastewaters in the literature [1,2,33,34].

The industrial wastewater influent in TP-4 usually undergoes (1) pre-treatment (screening grit and sieve) and (2) physicochemical treatment (coagulation tank, flocculation tank, and lamellar settling). This physicochemical treatment increases the dissolved concentration of Fe and Al in the final effluent due to the use of high amounts of aluminum and ferric salts as coagulants [12]. These metal coagulants are commonly used in wastewater treatment, not only for their effectiveness but also for their availability and relatively low cost [35].

The levels of Mn detected at this plant could be related to the influents (inputs) of the glass facility or the industries of paint, varnishes, colorants, etc., in which Mn (or its salts) are widely used [36].

Finally, the high concentration of the other dissolved metals analyzed in the effluent of the TP-4 facility is consistent with the reported values in the literature. Zn levels (114.816 ± 28.488 μg·L^−1^) are comparable to those reported by [34] (160 ± 30 µg·L^−1^) and [1] (223 μg·L^−1^) in final effluents of WWTP receiving mostly industrial wastewaters. The same applies to Ni (18.044 ± 3.930 μg·L^−1^), which is in the same order of magnitude as the values reported in [1] (11.7 µg·L^−1^). However, the concentration of Cu and Pb in TP-4 was lower than those reported in [31] (180 µg·L^−1^ and 190 µg·L^−1^ for Cu and Pb, respectively) and [1] (20.8 µg·L^−1^ and 2.5 µg·L^−1^ for Cu and Pb, respectively). The input from specific facilities that use these elements in their industrial processes (i.e., lead-acid battery factories) caused the high presence of Cu and Pb reported in the literature. This kind of industry does not affect the TP-4 facility.

In the final discharges of the other facilities, where inlets are different and diverse, Cd concentrations were low (<0.0015 μg·L^−1^) in all cases, whereas Ni, Pb, Cr, Cu, and Co, eventually peaked in some of them (i.e., Cu in TP-3). On the other hand, Zn and Mn were abundant (ranging between 5.160–31.492 μg·L^−1^ and 1.211–40.109 μg·L^−1^, respectively), and Al and Fe were clearly predominant, reaching high concentrations at times (ranging between 0.786–70.619 µg·L^−1^ and 0.2999–111.299 µg·L^−1^, respectively). The highest levels in Al and Fe were registered in the effluents of those facilities in which large amounts of aluminum and ferrous salts are commonly used as coagulants in their treatments (i.e., the TP-2 facility).

Altogether, the mean concentrations of the total dissolved metals in the effluents of the studied facilities, measured by spot sampling, ranged broadly. Thus, the DGT technique arises as a proper tool for effluents monitoring. In addition, we found high variability in the metal content over the sampling time, except for TP-5 (the power plant) and TP-6 (the aquaculture facility). Differences in the relative metal load contribution from different sources (industrial, domestic, or mixed areas) such as pipelines and taps, wastewaters from washing the streets and roads (due to consumption of automotive parts such as tires, brakes, etc.), combustion of fuel, activities and services (car washes, dentists, hospitals), some industries, etc. [1,34,37] may be the main cause of this variability. On the contrary, even though rain events usually lead to high suspended solids concentrations, with high concentrations of metals in the WWTP, the stormwater runoff is not related to the high levels found here, as the sampling was carried out after a long dry period.

As expected, in this study, we found higher metal contents in those WWTPs with larger industrial contributions in their inlets, such as TP-4 (industrial wastewaters), TP-2 (mixed wastewaters), TP-1 (household wastewaters), and TP-3 (mixed wastewaters).

However, we cannot provide a compliance assessment as there are no regional threshold values (limits) for the levels of metals in the effluents discharged into the sea.

#### 3.2.2. Dissolved Labile Metals

Results in Table 2 for DGT-labile metals are coherent with those reported over a wide range of solution conditions [21,38]. We assessed DGT-labile metals in every sampling site, except for Cu in the TP-4 facility. Here, we did not observe any difference between the exposed DGTs and the laboratory blanks for this metal, so this result was not taken into consideration.

Predictably, the metals with the highest concentrations in the total dissolved fraction, i.e., Zn, Fe, and Mn, were also those with the highest concentration in the labile fraction.

Note that concentrations of the DGT-measured labile metals shown in Table 2 are the mean values of the 3 exposed replicates, whereas, in the total dissolved fraction, results are the mean values of 3 spot-samples that represented temporal variations during the DGT-deployment. DGT-labile concentrations varied at these ranges (all expressed in μg·L^−1^ units): Cd [0.001–0.008], Ni [0.376–7.610], Pb [0.017–0.047], Cr [0.139–0.465], Cu [0.041–0.791], Zn [1.361–19.943], Fe [3.070–6200.034], Mn [1.306–66.146], and Co [0.024–2.372].

Furthermore, we observed the highest concentrations of most of the labile metals in the TP-4 facility, except for four metals: Cd, Cu, Pb, and Mn. The highest contents in Cd and Cu were measured at TP-6, the marine fish farm. The discharge from TP-1, the largest studied WWTP, registered the highest values in Pb. Finally, the highest levels in Mn were found at TP-2, a mixed domestic and industrial WWTP.

#### 3.2.3. Comparison between the Concentrations of the Total-Dissolved and the Labile Metal Fractions

As expected, differences were observed when comparing results from the 2 sampling techniques used in this study (Table 2): the DGT-labile 4-day time-integrated concentrations (average of the triplicates) were lower compared to the total dissolved concentrations measured in discrete water samples (day 0, 2, and 4) in almost every sampled effluent, which indicates that these devices accumulate only a limited fraction of the total metal.

The dissolved fraction comprises the free metal ions, labile inorganic and organic complexes, as well as inert high molecular organic metal complexes and colloids [39]. DGT results may provide valuable information on this speciation in the sampled discharges since they are the measurement of free ions and labile organic/inorganic complexes, and they provide information regarding the concentration that is considered potentially bioavailable. However, due to the complexity of the characteristics of the different sampled effluents, the speciation of trace metals is very challenging in this short-term study.

Therefore, direct comparison between the concentration measured by spot samples and the concentration measured by the DGT technique is not possible [20,24], and when explaining these results, the following aspects should be considered:

- Differences in the fraction measured in spot sampling (total dissolved) and by DGTs (dissolved labile). As mentioned before, different chemical forms are measured depending on the fraction considered. The concentrations found of total dissolved metals tended to be generally higher than the DGT-labile concentrations, so the percentage of the labile fraction being part of the total dissolved fraction is normally less than 100% (Table 2). Although, some exceptions were observed, mainly in the TP-6 facility, where the percentage of the labile fraction per the total dissolved concentration exceeded 100% in most of the studied metals except for Cd, Ni, and Zn (Supplementary Information S3).
- Differences in the timescale of the spot-sampling measurements and the DGTs measurements. Results do not represent the same sampling timescale (Table 2). Total dissolved metal concentrations are the average of the metal concentrations measured at three specific times (day 0, 2, and 4), whereas the DGT provides 4 days-weighted average metal concentrations. Thus, the spot sampling can miss some peaks and/or decreases in metal concentrations and may not properly monitor the wide variation in the total dissolved metal content. The advantage of using DGT devices is their ability to measure time-weighted average concentrations over the deployment period providing more representative results for highly variable systems. This seems to be especially relevant in the case of the marine fish farm facility (TP- 6), where differences in the temporal distribution of the farming processes (feeding, use of chemicals, water recirculation, waste load, etc.) may affect the temporal content and speciation of metals significantly [40].
- Differences in the physicochemical characteristics of the analyzed effluents. The physicochemical conditions may impact the forms’ distribution (speciation) for a given metal. In this study, we determined the physicochemical parameters in the different wastewater effluents at each sampling day (Supplementary Information S4). The overall temperature in the 6 outlets ranged between 21.9 and 29.0 °C. The highest values in temperature were measured at TP-3 (with domestic and industrial influents), whereas the lowest temperatures were registered at the marine fish farm (TP-6). The pH values ranged between 4.73, measured in the TP-4 effluent (treating industrial influents by flocculation), and 7.73, in TP-3 (with mixed domestic and industrial influents). Furthermore, the lowest dissolved oxygen values were registered at the TP-4 facility (0.36 mg·L^−1^), while, at the other facilities, the dissolved oxygen ranged between 6.19 and 7.31 mg·L^−1^. In addition, as some of the sampled effluents’ water source was seawater (TP-5 cooling water and TP-6 marine fish farm), the overall recorded conductivity (25 °C) ranged widely, between 1.66 and 55.67 mS·cm^−1^.

The mobility, bioavailability, and toxicity of heavy metals depend on their speciation rather than on their total concentrations in water. Some of the main physicochemical parameters that determine the bioavailability of metal species in wastewater effluents are the DOC (by complexation with dissolved organic matter forming organometallic complexes) and SPM (by association with suspended particles), which can markedly reduce the free ion concentration of the metal [18,19,41].

DOC, SPM, and Turb measured in the spot samples from the TP-4 discharge (industrial wastewater) exceeded by two orders of magnitude those determined in the effluents from the other facilities. In TP-4, these parameters reached averaged values of 753.43 mg L^−1^, 458.67 mg L^−1^, and 435 FNU, respectively. Even though in this study the relationship (correlation) between these parameters and metal speciation has not been addressed due to the limited data availability, the high concentrations of DOC in all the samples of TP-4 facility may explain the low % of the labile fraction per the total dissolved fraction in most of the studied metals. In fact, in the other facilities with a much lower range in DOC, [0.5–15.2] mg·L^−1^, the % of the labile fraction is higher, especially in the conventional WWTPs (Table 2). Further research on the influence of the physicochemical temporal variability in wastewater metal speciation should be developed, increasing the spot sampling frequency according to the daily variability in each effluent. In addition, the relationship between other physicochemical parameters, such as pH, conductivity, alkalinity, and the presence of other cations and anions in each effluent should be assessed too.

### 3.3. Effluent Discharge Impact on Coastal Water Bodies

Effective reduction of heavy metals loads discharged into receiving waters and mitigation of consequent environmental negative impacts require knowledge about their sources and emissions, as shown by the results of this study. The Environment Agencies usually set numerical limits on the content of pollutants in effluents. Thus, they consider both those parameters that the discharge would probably contain and the defined Environmental Quality Standard (EQS) to assess the chemical status of the water bodies. In addition, EQSs represent a target to reach when addressing risk management measures for specific pollutants and, in particular, concerning the reduction of emissions (EQS EC Guidance, 2018) [42]. As explained before, EQS values, defined in the updated WFD, were transposed to the Spanish legal framework by the RD 817/2015 [10].

**Figure 2:**
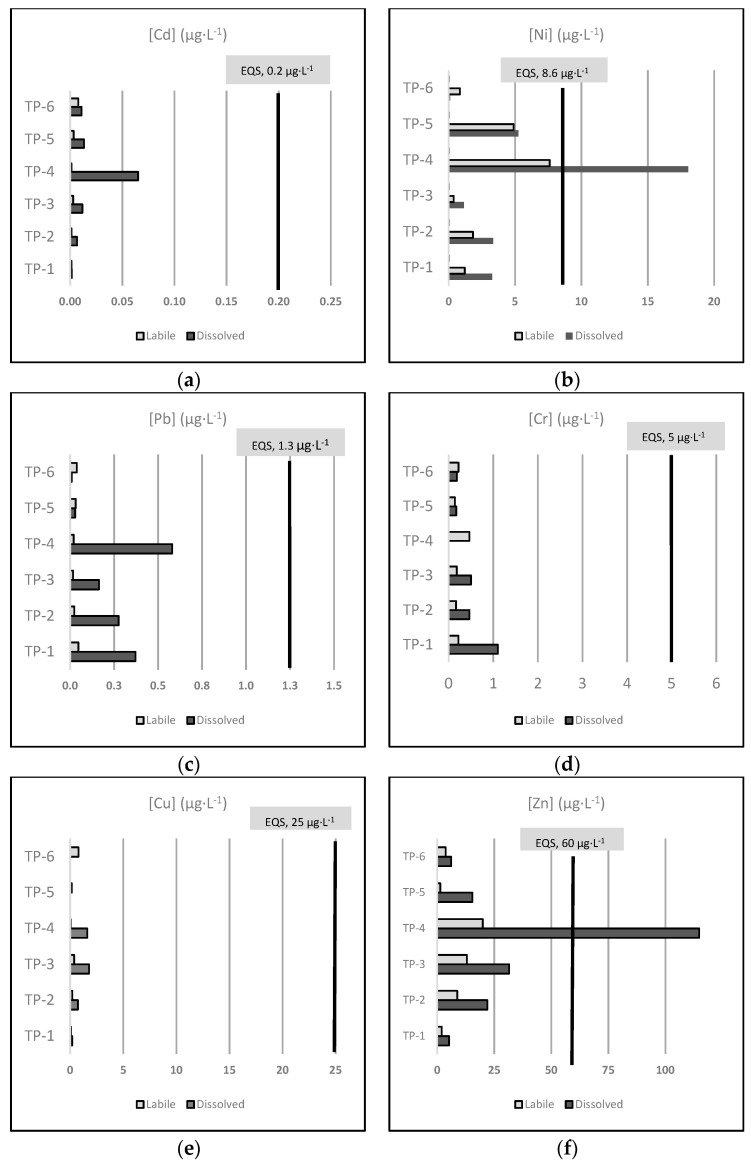
Mean concentrations (µg·L^−1^) of the six regulated metals included in this study, measured as the dissolved and labile fraction in all the sampled effluents, with the indication of the AA-EQS values for other surface waters of each metal: Cd (**a**), Ni (**b**), Pb (**c**), Cr (**d**), Cu (**e**), and Zn (**f**). Note: all Cr is considered as Cr VI according to the precautionary principle.

Here, we considered the AA-EQS established in the Spanish regulation to assess the total dissolved metal concentrations in the effluents in terms of contribution to the EQS exceedance within the receiving water body (Figure 2).

Even though suitable EQS are not currently established for the labile fraction, we also include the labile metal concentrations in Figure 2 for informational purposes only. To define proper EQS for the labile fraction, a recently finished EU-funded project named MONITOOL [43] has proposed specific environmental objectives for the passive-sampler measurement (EQS_DGT_) of Cd, Ni, and Pb in transitional and coastal waters.

Figure 2 represents the mean concentration (µg·L^−1^) of the six regulated metals included in this study (Cd, Ni, Pb, Cr, Cu, and Zn) measured in the dissolved and labile fraction of all the sampled effluents, regarding the established AA-EQS values for other surface waters of each metal. Note that we assume that all Cr is Cr VI, according to the precautionary principle.

As shown in Figure 2, only the average dissolved concentrations of Ni (18.044 µg·L^−1^) and Zn (114.815 µg·L^−1^), both in the effluent of the TP-4 facility, clearly exceeded the AA-EQS established for other surfaces waters (8.6 and 60 µg·L^−1^, respectively). These results show that the TP-4 industrial wastewater treatment was not efficient enough to diminish the concentration of toxic heavy metals like Ni and Zn, resulting in an anthropogenic pressure for the receiving waterbody.

In this case, these high metal concentrations in the effluent will probably be reduced in the receiving coastal water body due to the mixing processes and wastewater plume dilution through the submarine outfall [44,45], resulting in compliance with the AA-EQS for these metals. However, according to the established principles in the EU water policy (the precautionary principle, the preventive principle, and the principle that environmental damage should, as a priority, be rectified at the source), better control of the industrial sources discharging to the sewer is needed to minimize the income of highly contaminated wastewater into the WWTP. All industries are required to have a pre-treatment system for their wastewater by law.

We cannot provide a compliance assessment as there are no regional (Canarian) threshold values for the levels of metals in the effluents discharged into coastal waters. However, for reference purposes, comparing the average concentrations of the 10 total dissolved metals shown in Table 2 with the threshold limits (ELV range values) of the 3 Spanish regions mentioned above, only the average dissolved concentrations of Fe (9855.652 μg·L^−1^) in the effluent of the TP-4 facility, clearly exceeded this range established for discharges to coastal waters [2000–3600 μg·L^−1^]. None of the concentrations of the other nine metals measured in the effluents of the facilities sampled in this study exceeded the strictest value of the threshold established by these three regulations.

Furthermore, to address the contributions of the total dissolved and the labile metal fractions discharged from the facilities under study, we estimated the daily specific load for each metal and each facility (except for the TP-6, in which the flow data is not available) using the approach given by Equation (3):

(3) $$\mathrm{Load}=\overset{n}{\underset{i=1}{\sum }}\mathrm{ci}\;\mathrm{qi}\;\mathrm{ti}$$

where:

- ci is the mean concentration (total dissolved or labile) of each metal in the i^th^ sample measured in this study for each facility
- qi is the daily average flow from each facility, and ti is the time interval (1 day).

The results of this estimation are shown in Table 3.

The daily fluxes of dissolved and labile metals discharged by the TP-5 and TP-4 facilities calculated in this study (Table 3) show that the effluents from these two facilities specifically contribute to a final discharge of polymetallic flows into coastal waters. TP-5 facility presents relatively high daily loads for all metals (except for Fe) due to the large volumes discharged. In power plants like the one studied, cooling systems are the most water-intensive part (in this case, based on seawater) of the thermoelectric generation process [46]. Thus, the high daily flow of effluent (27,500 m^3^/h) leads to high daily loads of metals. Here, loads greater than 1 kg/day of total dissolved or labile, respectively, were calculated: 3466 and 3225 g/day of Ni; 10,170 and 898 g/day of Zn, and 1042 and 862 g/day of Mn. Total dissolved aluminum was 1897 g/day.

On the contrary, the TP-4 facility, which treats exclusively industrial wastewaters, reported the lowest daily flow of the studied facilities (30 m^3^/h). However, as a consequence of the high values of some of the metals measured in its effluent, high daily loads (total dissolved and labile) were observed for Fe (7096 total dissolved and 4464 labile g/day), Al (502 total dissolved g/day), and Mn (463 total dissolved and 12 labile g/day).

## 4. Conclusions

The mean concentration of the total dissolved and labile metals found in the studied effluents is dispersed in a broad range and shows high variability. Higher metal contents were found in the discharges of those facilities corresponding to WWTPs in decreasing order as: TP-4 > TP-2 > TP-1 > TP-3.

The highest values of mean total dissolved concentration of Zn, Fe, Mn, and Al were recorded at the TP-4 facility, which exclusively receives highly loaded industrial wastewater in which Al and Fe salts are added in the treatment. The same behavior was observed for these metals in the labile fraction recorded with the DGTs except for Mn and Al. For this facility, measures for reducing metal contaminations based on better control of the industrial intakes and also on the improvement in the treatments for heavy metals removal would be necessary. These measurements should be coherent with the pending establishment of regional regulatory limits for the levels of metals in the discharges into the sea.

We present the results of a study performed only in the discharged effluents. A complete assessment of the impact of metal discharge to coastal marine environment implies the use of an integrated monitoring approach, and these results should have been supported, at least, by the concentrations of such metals in the water column and sediment, but this information could not be obtained.

The limitations of low-frequency spot sampling, such as the lack of representativeness in dynamic systems such as discharges, can be compensated with the inclusion of complementary methodologies, such as DGTs, which integrate the system’s metal fluctuations and measure its labile fraction. The labile fraction is easily related to the ecotoxicological effects, improving the quality of the assessment. Besides, the improvement in the knowledge about the speciation of heavy metals is relevant to designing appropriate removal technologies.

The present results confirm the robustness of the DGT technique and its viability to measure dissolved labile metals when directly applied on different wastewater effluents, as DGT behaves predictably over a wide range of physicochemical conditions.

## Figures and Tables

**Table 1:** Main treatments and characteristics of the sampled facilities.

Label	Type of Facility ^1^	Wastewater Source	Size (p.e.) ^2^	Main Treatment	Discharge Flow (m^3^/h)
TP-1	WWTP	Household	600,000	Pre-treatment/Settling Activated sludge Chlorination	1100
TP-2	WWTP	Household + industry (10%)	171,600	Pre-treatment/Settling Activated sludge Chlorination	345
TP-3	WWTP	Household + industry (25%)	50,000	Pre-treatment Membrane bioreactor (MBR) Chlorination	136
TP-4	WWTP	Industry	4600	Pre-treatment Coagulation–flocculation settling	30
TP-5	TPP	Industry (cooling water of a thermal power plant)	No data	Aeration tanks/Settling	27,500
TP-6	MFF	Aquaculture (indoor marine fish-farm)	No data	Settling	75

^1^ WWTP: wastewater treatment plant; TPP: thermal power plant; MFF: marine fish farm. ^2^ p.e.: population equivalent.

**Table 2:** Concentrations of total dissolved (day 0, day 2, day 4 and mean ± SD values in µg·L^−1^) and dissolved labile metals (mean values of the 3 replicates in µg·L^−1^) determined in the final effluent of each facility. The percentage (%) of the labile dissolved fraction per the total dissolved is also shown for every metal.

Metal	Fraction	Facility					
		TP-1	TP-2	TP-3	TP-4	TP-5	TP-6
Cd	Dissolved, day 0	0.002	0.005	0.009	0.090	0.010	0.010
	Dissolved, day 2	0.002	0.010	0.013	0.088	0.017	0.012
	Dissolved, day 4	0.001	0.004	0.013	0.017	0.013	0.010
	Dissolved (mean ± SD)	0.002 ± 0	0.007 ± 0.003	0.012 ± 0.002	0.065 ± 0.041	0.013 ± 0.004	0.011 ± 0.001
	Labile	0.001	0.001	0.003	0.001	0.003	0.008
	% Labile	80	18	25	2	25	71
Ni	Dissolved, day 0	2.242	2.820	1.104	18.319	3.700	0.146
	Dissolved, day 2	3.665	4.304	1.157	21.829	2.707	0.117
	Dissolved, day 4	3.939	2.904	1.169	13.984	9.347	0.069
	Dissolved (mean ± SD)	3.282 ± 0.911	3.343 ± 0.833	1.143 ± 0.035	18.044 ± 3.930	5.251 ± 3.581	0.011 ± 0.001
	Labile	1.214	1.831	0.376	7.61	4.886	0.008
	% Labile	37	55	33	42	93	71
Pb	Dissolved, day 0	0.350	0.219	0.205	0.901	0.009	0.009
	Dissolved, day 2	0.377	0.353	0.130	0.764	0.067	0.009
	Dissolved, day 4	0.387	0.254	0.157	0.076	0.009	0.009
	Dissolved (mean ± SD)	0.371 ± 0.019	0.276 ± 0.069	0.164 ± 0.038	0.580 ± 0.442	0.028 ± 0.033	0.009 ± 0
	Labile	0.047	0.024	0.017	0.02	0.031	0.037
	% Labile	13	9	10	3	>100	>100
Cr	Dissolved, day 0	0.786	0.447	0.500	4.296	0.133	0.196
	Dissolved, day 2	1.207	0.582	0.503	4.730	0.170	0.190
	Dissolved, day 4	1.312	0.360	0.498	1.612	0.212	0.167
	Dissolved (mean ± SD)	1.102 ± 0.278	0.463 ± 0.112	0.5 ± 0.003	3.546 ± 1.689	0.172 ± 0.040	0.185 ± 0.015
	Labile	0.218	0.165	0.181	0.465	0.139	0.222
	% Labile	20	36	36	13	81	>100
Cu	Dissolved, day 0	0.242	0.438	2.300	2.385	0.015	0.015
	Dissolved, day 2	0.135	1.330	1.666	1.998	0.015	0.015
	Dissolved, day 4	0.182	0.403	1.356	0.440	0.015	0.015
	Dissolved (mean ± SD)	0.186 ± 0.054	0.724 ± 0.526	1.774 ± 0.481	1.608 ± 1.03	0.015 ± 0	0.015 ± 0
	Labile	0.068	0.196	0.38	nd	0.146	0.791
	% Labile	36	27	21	-	>100	>100
Zn	Dissolved, day 0	5.328	19.201	30.643	125.712	20.115	6.518
	Dissolved, day 2	5.249	26.584	31.016	136.248	21.053	6.413
	Dissolved, day 4	4.903	20.147	32.818	82.488	5.059	5.294
	Dissolved (mean ± SD)	5.160 ± 0.226	21.977 ± 4.017	31.492 ± 1.163	114.816 ± 28.488	15.409 ± 8.976	6.075 ± 0.679
	Labile	1.92	8.799	12.997	19.943	1.361	3.675
	% Labile	37	40	41	17	9	60
Al	Dissolved, day 0	29.823	72.254	17.724	911.029	0.861	0.861
	Dissolved, day 2	32.419	44.602	20.245	1056.066	0.861	0.748
	Dissolved, day 4	32.103	95.000	21.680	124.072	6.898	0.748
	Dissolved (mean ± SD)	31.448 ± 1.417	70.619 ± 25.238	19.883 ± 2.003	697.056 ± 501.489	2.874 ± 3.486	0.786 ± 0.065
	Labile	ND	ND	ND	ND	ND	ND
	% Labile	-	-	-	-	-	-
Fe	Dissolved, day 0	101.001	58.643	59.049	13,048.876	0.207	0.482
	Dissolved, day 2	114.755	108.238	52.918	11,627.790	0.207	0.207
	Dissolved, day 4	118.142	73.133	56.386	4890.290	1.118	0.207
	Dissolved (mean ± SD)	111.299 ± 9.078	80.005 ± 25.501	56.118 ± 3.074	9855.652 ± 4358.438	0.511 ± 0.526	0.299 ± 0.159
	Labile	18.37	15.171	5.972	6200.034	3.07	4.575
	% Labile	17	19	11	63	>100	>100
Mn	Dissolved, day 0	30.919	14.048	1.328	829.592	1.335	1.234
	Dissolved, day 2	32.832	62.795	0.223	718.726	1.432	1.283
	Dissolved, day 4	35.490	43.483	3.285	381.830	1.971	1.116
	Dissolved (mean ± SD)	33.080 ± 2.295	40.109 ± 24.548	1.612 ± 1.550	643.383 ± 233.195	1.579 ± 0.342	1.211 ± 0.086
	Labile	43.479	66.146	6.694	17.129	1.306	3.043
	% Labile	>100	>100	>100	3	83	>100
Co	Dissolved, day 0	0.251	0.613	0.283	7.344	0.360	0.007
	Dissolved, day 2	0.649	0.866	0.299	8.488	0.247	0.006
	Dissolved, day 4	0.646	0.544	0.321	4.146	1.049	0.008
	Dissolved (mean ± SD)	0.515 ± 0.229	0.674 ± 0.17	0.301 ± 0.019	6.659 ± 2.251	0.552 ± 0.434	0.007 ± 0.001
	Labile	0.033	0.116	0.024	2.372	0.498	0.057
	% Labile	6	17	8	36	90	>100

nd: not detected ND: not determined. DGT-labile concentrations for Al are not considered, as Chelex 100-DGT is not the most suitable adsorbent for this measurement [31,32].

**Table 3:** Estimation of the daily specific loads (g/day) of total dissolved and dissolved labile metals for the facilities under study.

Metal	Fraction	Facility					
		TP-1	TP-2	TP-3	TP-4	TP-5	TP-6
Cd	Total dissolved	0.040	0.055	0.038	0.047	8.735	0.020
	Dissolved labile	0.032	0.010	0.009	0.001	2.188	0.014
Ni	Total dissolved	86.639	27.680	3.732	12.992	3465.770	0.199
	Dissolved labile	32.041	15.164	1.226	5.479	3224.948	1.518
Pb	Total dissolved	9.801	2.282	0.535	0.418	18.563	0.016
	Dissolved labile	1.241	0.196	0.054	0.014	20.527	0.067
Cr	Total dissolved	29.086	3.835	1.633	2.553	113.199	0.331
	Dissolved labile	5.753	1.364	0.592	0.335	91.451	0.400
Cu	Total dissolved	4.919	5.994	5.790	1.157	9.900	0.027
	Dissolved labile	1.790	1.626	1.239	n.a.	96.509	1.423
Zn	Total dissolved	136.217	181.973	102.791	82.667	10,169.972	10.935
	Dissolved labile	50.686	72.855	42.421	14.359	898.025	6.615
Al	Total dissolved	830.235	584.723	64.897	501.880	1896.635	1.415
	Dissolved labile	n.a.	n.a.	n.a.	n.a.	n.a.	n.a.
Fe	Total dissolved	2938.306	662.441	183.168	7096.069	337.149	0.538
	Dissolved labile	484.962	125.615	19.494	4464.025	2025.898	8.234
Mn	Total dissolved	873.325	332.102	5.262	463.236	1042.385	2.180
	Dissolved labile	1147.854	547.690	21.850	12.333	861.648	5.478
Co	Total dissolved	13.603	5.581	0.982	4.795	364.386	0.013
	Dissolved labile	0.874	0.964	0.079	1.708	328.592	0.102

n.a.: not applicable.

## Supplementary Materials

The following are available online at https://www.mdpi.com/article/10.3390/ijerph182111582/s1, Supplementary Information S1: DGT-deployment, Supplementary Information S2: Laboratory blanks control; Supplementary Information S3: Labile-dissolved metal comparison at TP-6, Supplementary Information S4: Physicochemical parameters.
