# Supplementary material for: Inputs of Total and Labile Dissolved Metals from Six Facilities Continuously Discharging Treated Wastewaters to the Marine Environment of Gran Canaria Island (Canary Islands, Spain)
Source: Int J Environ Res Public Health. 2021 Nov 4;18(21):11582. doi: 10.3390/ijerph182111582 (PMC8583637; doi:10.3390/ijerph182111582)
Supplement: Supplementary file 1 [file ijerph-18-11582-s001.zip › Supplementary information S2 (Tables S1.1 to S1.9) Lab blank controls.pdf]

## Supplementary Information S2:

DGT laboratory blank control calculations consisted in establishing the existence of significant differences among the mean metal concentrations measured in the DGT laboratory blanks and the mean metal concentration accumulated in DGTs after exposure of 4 complete days. For that purpose, the independent samples t-test (Student test) was applied for each metal at each sampling sites. The significance level was set at $\alpha$=0.05, corresponding to a confidence level of 95%. A value of $P < 0.05$ means that mean concentrations in DGT exposed (D4) is significantly greater than mean concentration found in DGT lab blanks.

These analyses were carried out by the open source Jamovi software (www.jamovi.org), version 1.6.9. The tables below (Table S1 to S9) summarize the results of the test for each pair (DGT exposed (D4) and DGT lab blank) grouped by metal.

**Table S1.1** Results of the Student test for Cd for each sampled facility.

| Facility | P-value | Result |
|---|---|---|
| TP-1 | < 0.001 | [DGT exposed (D4)] > [ DGT lab blank] |
| TP-2 | < 0.001 | [DGT exposed (D4)] > [ DGT lab blank] |
| TP-3 | < 0.001 | [DGT exposed (D4)] > [ DGT lab blank] |
| TP-4 | 0.003 | [DGT exposed (D4)] > [ DGT lab blank] |
| TP-5 | < 0.001 | [DGT exposed (D4)] > [ DGT lab blank] |
| TP-6 | < 0.001 | [DGT exposed (D4)] > [ DGT lab blank] |

**Table S1.2.** Results of the Student test for Ni for each sampled facility.

| Facility | P-value | Result |
|---|---|---|
| TP-1 | < 0.001 | [DGT exposed (D4)] > [ DGT lab blank] |
| TP-2 | < 0.001 | [DGT exposed (D4)] > [ DGT lab blank] |
| TP-3 | < 0.001 | [DGT exposed (D4)] > [ DGT lab blank] |
| TP-4 | < 0.001 | [DGT exposed (D4)] > [ DGT lab blank] |
| TP-5 | < 0.001 | [DGT exposed (D4)] > [ DGT lab blank] |
| TP-6 | < 0.001 | [DGT exposed (D4)] > [ DGT lab blank] |

**Table S1.3.** Results of the Student test for Pb for each sampled facility.

| Facility | P-value | Result |
|---|---|---|
| TP-1 | < 0.001 | [DGT exposed (D4)] > [ DGT lab blank] |
| TP-2 | < 0.001 | [DGT exposed (D4)] > [ DGT lab blank] |
| TP-3 | < 0.001 | [DGT exposed (D4)] > [ DGT lab blank] |
| TP-4 | 0.002 | [DGT exposed (D4)] > [ DGT lab blank] |
| TP-5 | < 0.001 | [DGT exposed (D4)] > [ DGT lab blank] |

| | | |
|---|---|---|
| TP-6 | < 0.001 | [DGT exposed (D4)] > [ DGT lab blank] |

**Table S1.4.** Results of the Student test for Cr for each sampled facility.

| Facility | P-value | Result |
|---|---|---|
| TP-1 | < 0.001 | [DGT exposed (D4)] > [ DGT lab blank] |
| TP-2 | 0.003 | [DGT exposed (D4)] > [ DGT lab blank] |
| TP-3 | < 0.001 | [DGT exposed (D4)] > [ DGT lab blank] |
| TP-4 | 0.002 | [DGT exposed (D4)] > [ DGT lab blank] |
| TP-5 | 0.005 | [DGT exposed (D4)] > [ DGT lab blank] |
| TP-6 | < 0.001 | [DGT exposed (D4)] > [ DGT lab blank] |

**Table S1.5.** Results of the Student test for Cu for each sampled facility.

| Facility | P-value | Result |
|---|---|---|
| TP-1 | 0.002 | [DGT exposed (D4)] > [ DGT lab blank] |
| TP-2 | < 0.001 | [DGT exposed (D4)] > [ DGT lab blank] |
| TP-3 | < 0.001 | [DGT exposed (D4)] > [ DGT lab blank] |
| TP-4 | 0.123 | [DGT exposed (D4)] ≤ [ DGT lab blank] |
| TP-5 | 0.005 | [DGT exposed (D4)] > [ DGT lab blank] |
| TP-6 | < 0.001 | [DGT exposed (D4)] > [ DGT lab blank] |

**Table S1.6**. Results of the Student test for Zn for each sampled facility.

| Facility | P-value | Result |
|---|---|---|
| TP-1 | < 0.001 | [DGT exposed (D4)] > [ DGT lab blank] |
| TP-2 | < 0.001 | [DGT exposed (D4)] > [ DGT lab blank] |
| TP-3 | < 0.001 | [DGT exposed (D4)] > [ DGT lab blank] |
| TP-4 | < 0.001 | [DGT exposed (D4)] > [ DGT lab blank] |
| TP-5 | < 0.001 | [DGT exposed (D4)] > [ DGT lab blank] |
| TP-6 | < 0.001 | [DGT exposed (D4)] > [ DGT lab blank] |

**Table S1.7.** Results of the Student test for Fe for each sampled facility.

| Facility | P-value | Result |
|---|---|---|
| TP-1 | < 0.001 | [DGT exposed (D4)] > [ DGT lab blank] |
| TP-2 | < 0.001 | [DGT exposed (D4)] > [ DGT lab blank] |
| TP-3 | < 0.001 | [DGT exposed (D4)] > [ DGT lab blank] |
| TP-4 | < 0.001 | [DGT exposed (D4)] > [ DGT lab blank] |
| TP-5 | < 0.001 | [DGT exposed (D4)] > [ DGT lab blank] |
| TP-1 | < 0.001 | [DGT exposed (D4)] > [ DGT lab blank] |

**Table S1.8.** Results of the Student test for Mn for each sampled facility.

| Facility | P-value | Result |
|---|---|---|
| TP-1 | < 0.001 | [DGT exposed (D4)] > [ DGT lab blank] |
| TP-2 | < 0.001 | [DGT exposed (D4)] > [ DGT lab blank] |
| TP-3 | < 0.001 | [DGT exposed (D4)] > [ DGT lab blank] |
| TP-4 | < 0.001 | [DGT exposed (D4)] > [ DGT lab blank] |
| TP-5 | < 0.001 | [DGT exposed (D4)] > [ DGT lab blank] |
| TP-6 | 0.002 | [DGT exposed (D4)] > [ DGT lab blank] |

**Table S1.9.** Results of the Student test for Co for each sampled facility.

| Facility | P-value | Result |
|---|---|---|
| TP-1 | < 0.001 | [DGT exposed (D4)] > [ DGT lab blank] |
| TP-2 | < 0.001 | [DGT exposed (D4)] > [ DGT lab blank] |
| TP-3 | < 0.001 | [DGT exposed (D4)] > [ DGT lab blank] |
| TP-4 | < 0.001 | [DGT exposed (D4)] > [ DGT lab blank] |
| TP-5 | < 0.001 | [DGT exposed (D4)] > [ DGT lab blank] |
| TP-6 | <0.001 | [DGT exposed (D4)] > [ DGT lab blank] |
