# Supplementary material for: Inputs of Total and Labile Dissolved Metals from Six Facilities Continuously Discharging Treated Wastewaters to the Marine Environment of Gran Canaria Island (Canary Islands, Spain)
Source: Int J Environ Res Public Health. 2021 Nov 4;18(21):11582. doi: 10.3390/ijerph182111582 (PMC8583637; doi:10.3390/ijerph182111582)
Supplement: Supplementary file 1 [file ijerph-18-11582-s001.zip › Supplementary information S3 Labile and total dissolved comparison TP_6.pdf]

Supplementary Information S3:

These calculations consisted in establishing the existence of significant differences among the total dissolved metal concentrations measured in the discrete samples (Day 0, 2 and 4)) and the labile metal concentration accumulated in DGTs after exposure of 4 complete days in TP-6 facility. For that purpose, the independent samples t-test (Student test) was applied for each metal The significance level was set at α=0.05, corresponding to a confidence level of 95%.

These analyses were carried out by the open source Jamovi software (www.jamovi.org), version 1.6.9. The table below, Table S12, summarizes the results of the test for each pairs by metal.

Table S2. Results of the Student test for labile and total dissolved metal concentration comparison in TP-6 facility for each metal

**Metal**	**P-value**	**Result**
Cd	0.992	[total dissolved metals] > [labile metals]
Ni	0.007	[labile metals] > [total dissolved metals]
Pb	< 0.001	[labile metals] > [total dissolved metals]
Cr	0.077	[total dissolved metals] > [labile metals]
Cu	<0.001	[labile metals] > [total dissolved metals]
Zn	0.994	[total dissolved metals] > [labile metals]
Fe	< 0.001	[labile metals] > [total dissolved metals]
Mn	0.01	[labile metals] > [total dissolved metals]
Co	0.011	[labile metals] > [total dissolved metals]
