# Supplementary material for: Inputs of Total and Labile Dissolved Metals from Six Facilities Continuously Discharging Treated Wastewaters to the Marine Environment of Gran Canaria Island (Canary Islands, Spain)
Source: Int J Environ Res Public Health. 2021 Nov 4;18(21):11582. doi: 10.3390/ijerph182111582 (PMC8583637; doi:10.3390/ijerph182111582)
Supplement: Supplementary file 1 [file ijerph-18-11582-s001.zip › Supplementary information S4 on-site physicochemical parameters .pdf]

Supplementary Information S4:

Table S3. Physicochemical parameters of each effluent spot sampling time across all sampling days. T, DO, EC and pH were recorded on-site with a calibrated multiparameter probe while DOC, SPM and Turb were determined at the laboratory in the collected spot samples.

Facility	Day of sampling	T (°C)	DO (% / mg·L^{-1})	EC (mS·cm^{-1})	pH (pH units)	DOC (mg·L^{-1})	SPM (mg·L^{-1})	Turb (FNU)
TP-1	Day 0	24.7	89.8 / 7.02	2.85	7.29	8.2	3	2.37
	Day 2	27.0	88.3 / 6.96	3.15	7.45	11.2	4	2.20
	Day 4	26.7	91.9 / 7.24	3.41	7.62	11.2	4	1.12
TP-2	Day 0	28.3	84.9 / 6.54	3.09	7.35	13.5	4	2.72
	Day 2	28.7	83.9 / 6.40	4.03	7.39	15.2	18	9.06
	Day 4	27.9	85.7 / 6.66	3.13	7.42	13.2	4	2.64
TP-3	Day 0	28.6	94.7 / 7.31	1.85	7.35	7.8	2	1.26
	Day 2	28.7	91.7 / 7.08	1.67	7.51	7.6	2	1.02
	Day 4	29.0	93.9 / 7.20	1.66	7.73	6.8	2	0.44
TP-4	Day 0	26.0	22.3 / 1.80	3.12	4.88	817.8	413	481
	Day 2	26.2	10.7 / 0.85	4.19	4.73	1188.0	740	668
	Day 4	24.9	4.5 / 0.36	2.91	5.24	254.5	223	156
TP-5	Day 0	27.9	102.9 / 6.64	52.05	6.81	1.9	18	10.90
	Day 2	28.0	97.0/ 6.19	55.50	6.96	1.2	6	2.91
	Day 4	26.6	101.2/ 6.60	55.60	6.79	< 0.5	6	3.11
TP-6	Day 0	22.2	101.8 / 7.12	55.67	7.38	2.0	2	0.27
	Day 2	22.7	103.1/ 7.16	55.54	7.24	1.1	3	0.89
	Day 4	21.9	102.0 / 7.19	55.55	7.19	1.5	3	0.61

T: temperature ; DO: dissolved oxygen; EC: electrical conductivity (25 °C); DOC: Dissolved Organic Carbon; SPM: suspended particulate matter; Turb: turbidity.
