# Supplementary figures and images for: Inputs of Total and Labile Dissolved Metals from Six Facilities Continuously Discharging Treated Wastewaters to the Marine Environment of Gran Canaria Island (Canary Islands, Spain)
Source: Int J Environ Res Public Health. 2021 Nov 4;18(21):11582. doi: 10.3390/ijerph182111582 (PMC8583637; doi:10.3390/ijerph182111582)

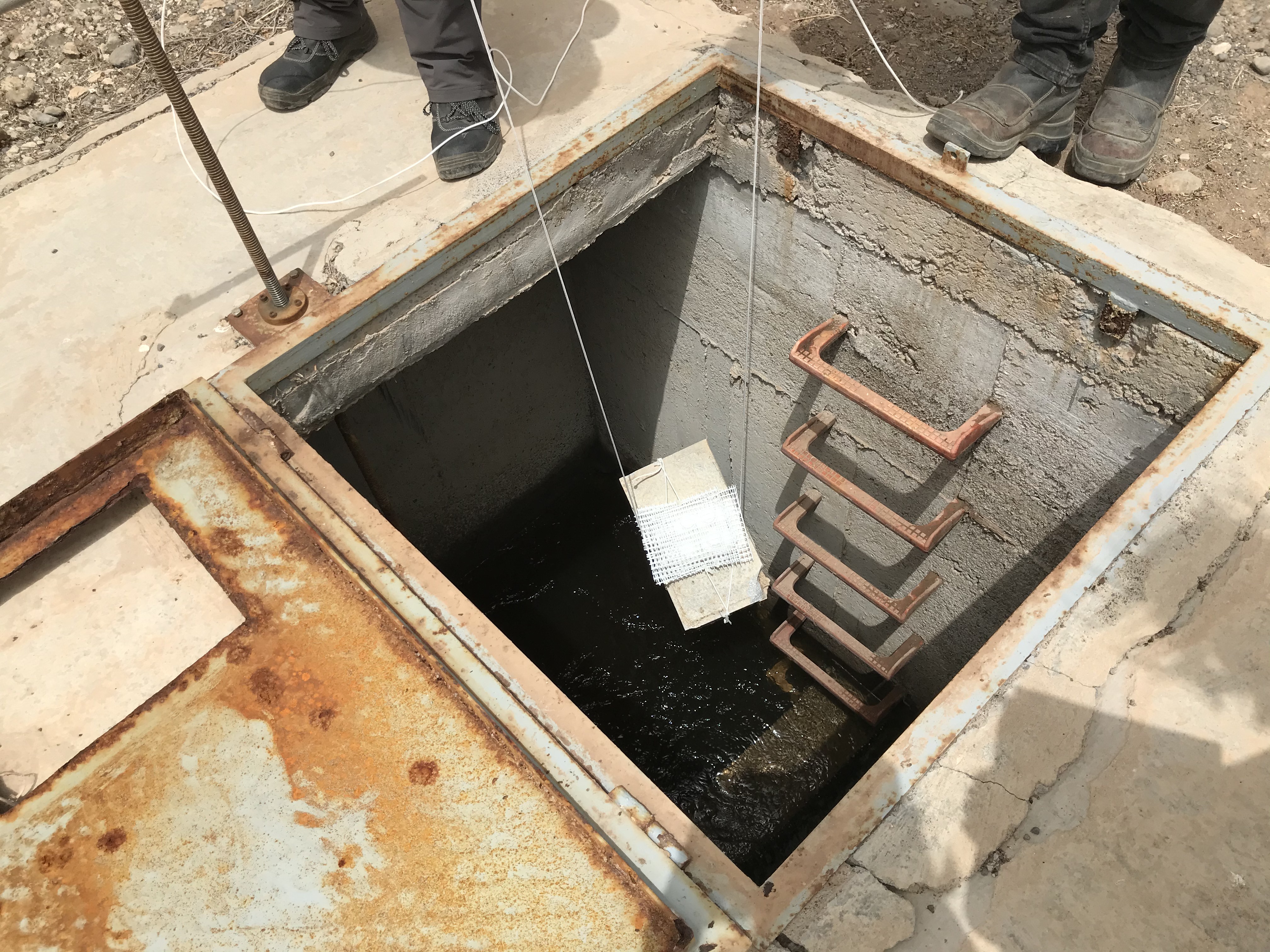

Supplement: Supplementary file 1 [file ijerph-18-11582-s001.zip › Supplementary information S1 Figure DGT deployment.JPG]
